# Supplementary material for: Pyruvate Dehydrogenase Kinase 1 inhibition mediated oxidative phosphorylation enhancement in cartilage promotes osteoarthritis progression
Source: BMC Musculoskelet Disord. 2023 Jul 20;24:597. doi: 10.1186/s12891-023-06585-6 (PMC10357736; doi:10.1186/s12891-023-06585-6)
Supplement: Supplementary file 1 — Additional file 1. [file 12891_2023_6585_MOESM1_ESM.zip › BP.Gsea.1653623667859/GOBP_REGULATION_OF_GLIAL_CELL_DIFFERENTIATION.html]

Details for gene set GOBP\_REGULATION\_OF\_GLIAL\_CELL\_DIFFERENTIATION[GSEA]

|  || Dataset | 55457\_collapsed\_to\_symbols.cls.cls #OA\_versus\_normal.cls.cls #OA\_versus\_normal\_repos |
| Phenotype | cls.cls#OA\_versus\_normal\_repos |
| Upregulated in class | normal |
| GeneSet | GOBP\_REGULATION\_OF\_GLIAL\_CELL\_DIFFERENTIATION |
| Enrichment Score (ES) | -0.4517428 |
| Normalized Enrichment Score (NES) | -1.7689046 |
| Nominal p-value | 0.0 |
| FDR q-value | 0.12104866 |
| FWER p-Value | 0.997 |
Table: GSEA Results Summary

  

Fig 1: Enrichment plot: GOBP\_REGULATION\_OF\_GLIAL\_CELL\_DIFFERENTIATION      
 Profile of the Running ES Score & Positions of GeneSet Members on the Rank Ordered List

  

| SYMBOL | TITLE | RANK IN GENE LIST | RANK METRIC SCORE | RUNNING ES | CORE ENRICHMENT || 1 | TREM2 | triggering receptor expressed on myeloid cells 2 [Source:HGNC Symbol;Acc:HGNC:17761] | 342 | 0.726 | 0.0086 | No |
| 2 | PTN | pleiotrophin [Source:HGNC Symbol;Acc:HGNC:9630] | 402 | 0.706 | 0.0382 | No |
| 3 | ZNF365 | zinc finger protein 365 [Source:HGNC Symbol;Acc:HGNC:18194] | 1390 | 0.444 | -0.0171 | No |
| 4 | WDR1 | WD repeat domain 1 [Source:HGNC Symbol;Acc:HGNC:12754] | 1851 | 0.379 | -0.0345 | No |
| 5 | OLIG2 | oligodendrocyte transcription factor 2 [Source:HGNC Symbol;Acc:HGNC:9398] | 1894 | 0.373 | -0.0197 | No |
| 6 | TNFRSF21 | TNF receptor superfamily member 21 [Source:HGNC Symbol;Acc:HGNC:13469] | 2228 | 0.336 | -0.0293 | No |
| 7 | TGFB1 | transforming growth factor beta 1 [Source:HGNC Symbol;Acc:HGNC:11766] | 2490 | 0.303 | -0.0349 | No |
| 8 | LIN28A | lin-28 homolog A [Source:HGNC Symbol;Acc:HGNC:15986] | 3140 | 0.236 | -0.0740 | No |
| 9 | DAG1 | dystroglycan 1 [Source:HGNC Symbol;Acc:HGNC:2666] | 3203 | 0.230 | -0.0676 | No |
| 10 | ASPA | aspartoacylase [Source:HGNC Symbol;Acc:HGNC:756] | 3610 | 0.192 | -0.0899 | No |
| 11 | CDK1 | cyclin dependent kinase 1 [Source:HGNC Symbol;Acc:HGNC:1722] | 3744 | 0.182 | -0.0914 | No |
| 12 | SPINT1 | "serine peptidase inhibitor, Kunitz type 1 [Source:HGNC Symbol;Acc:HGNC:11246]" | 3938 | 0.161 | -0.0986 | No |
| 13 | SOX1 | SRY-box transcription factor 1 [Source:HGNC Symbol;Acc:HGNC:11189] | 4065 | 0.150 | -0.1012 | No |
| 14 | F2 | "coagulation factor II, thrombin [Source:HGNC Symbol;Acc:HGNC:3535]" | 4745 | 0.092 | -0.1496 | No |
| 15 | SERPINE2 | serpin family E member 2 [Source:HGNC Symbol;Acc:HGNC:8951] | 4873 | 0.082 | -0.1555 | No |
| 16 | TP73 | tumor protein p73 [Source:HGNC Symbol;Acc:HGNC:12003] | 5222 | 0.055 | -0.1800 | No |
| 17 | MDK | midkine [Source:HGNC Symbol;Acc:HGNC:6972] | 5828 | 0.007 | -0.2267 | No |
| 18 | CXCR4 | C-X-C motif chemokine receptor 4 [Source:HGNC Symbol;Acc:HGNC:2561] | 6382 | -0.034 | -0.2681 | No |
| 19 | TENM4 | teneurin transmembrane protein 4 [Source:HGNC Symbol;Acc:HGNC:29945] | 6675 | -0.054 | -0.2883 | No |
| 20 | NR1D1 | nuclear receptor subfamily 1 group D member 1 [Source:HGNC Symbol;Acc:HGNC:7962] | 6974 | -0.078 | -0.3077 | No |
| 21 | DRD3 | dopamine receptor D3 [Source:HGNC Symbol;Acc:HGNC:3024] | 7101 | -0.088 | -0.3132 | No |
| 22 | PTPRZ1 | protein tyrosine phosphatase receptor type Z1 [Source:HGNC Symbol;Acc:HGNC:9685] | 7123 | -0.090 | -0.3105 | No |
| 23 | NR2E1 | nuclear receptor subfamily 2 group E member 1 [Source:HGNC Symbol;Acc:HGNC:7973] | 7660 | -0.132 | -0.3458 | No |
| 24 | DUSP10 | dual specificity phosphatase 10 [Source:HGNC Symbol;Acc:HGNC:3065] | 7775 | -0.140 | -0.3479 | No |
| 25 | PRMT5 | protein arginine methyltransferase 5 [Source:HGNC Symbol;Acc:HGNC:10894] | 7904 | -0.148 | -0.3507 | No |
| 26 | HDAC1 | histone deacetylase 1 [Source:HGNC Symbol;Acc:HGNC:4852] | 7911 | -0.148 | -0.3440 | No |
| 27 | TLR2 | toll like receptor 2 [Source:HGNC Symbol;Acc:HGNC:11848] | 7991 | -0.154 | -0.3427 | No |
| 28 | ID4 | "inhibitor of DNA binding 4, HLH protein [Source:HGNC Symbol;Acc:HGNC:5363]" | 8174 | -0.169 | -0.3487 | No |
| 29 | DAAM2 | dishevelled associated activator of morphogenesis 2 [Source:HGNC Symbol;Acc:HGNC:18143] | 8227 | -0.173 | -0.3443 | No |
| 30 | SHH | sonic hedgehog signaling molecule [Source:HGNC Symbol;Acc:HGNC:10848] | 8941 | -0.227 | -0.3888 | No |
| 31 | NKX2-2 | NK2 homeobox 2 [Source:HGNC Symbol;Acc:HGNC:7835] | 8977 | -0.230 | -0.3804 | No |
| 32 | DLX2 | distal-less homeobox 2 [Source:HGNC Symbol;Acc:HGNC:2915] | 9168 | -0.245 | -0.3833 | No |
| 33 | NF1 | neurofibromin 1 [Source:HGNC Symbol;Acc:HGNC:7765] | 9194 | -0.247 | -0.3733 | No |
| 34 | HDAC2 | histone deacetylase 2 [Source:HGNC Symbol;Acc:HGNC:4853] | 9359 | -0.260 | -0.3734 | No |
| 35 | MAG | myelin associated glycoprotein [Source:HGNC Symbol;Acc:HGNC:6783] | 9490 | -0.273 | -0.3703 | No |
| 36 | GPR37L1 | G protein-coupled receptor 37 like 1 [Source:HGNC Symbol;Acc:HGNC:14923] | 9519 | -0.276 | -0.3591 | No |
| 37 | BIN1 | bridging integrator 1 [Source:HGNC Symbol;Acc:HGNC:1052] | 10123 | -0.333 | -0.3898 | No |
| 38 | BMP2 | bone morphogenetic protein 2 [Source:HGNC Symbol;Acc:HGNC:1069] | 10919 | -0.420 | -0.4314 | Yes |
| 39 | KDM4A | lysine demethylase 4A [Source:HGNC Symbol;Acc:HGNC:22978] | 10936 | -0.423 | -0.4121 | Yes |
| 40 | TNFRSF1B | TNF receptor superfamily member 1B [Source:HGNC Symbol;Acc:HGNC:11917] | 10969 | -0.427 | -0.3939 | Yes |
| 41 | PRPF19 | pre-mRNA processing factor 19 [Source:HGNC Symbol;Acc:HGNC:17896] | 11269 | -0.467 | -0.3945 | Yes |
| 42 | NKX6-1 | NK6 homeobox 1 [Source:HGNC Symbol;Acc:HGNC:7839] | 11314 | -0.473 | -0.3750 | Yes |
| 43 | DAB1 | DAB adaptor protein 1 [Source:HGNC Symbol;Acc:HGNC:2661] | 11815 | -0.552 | -0.3871 | Yes |
| 44 | ID2 | inhibitor of DNA binding 2 [Source:HGNC Symbol;Acc:HGNC:5361] | 11852 | -0.560 | -0.3628 | Yes |
| 45 | CNTN2 | contactin 2 [Source:HGNC Symbol;Acc:HGNC:2172] | 11858 | -0.561 | -0.3359 | Yes |
| 46 | EPHA4 | EPH receptor A4 [Source:HGNC Symbol;Acc:HGNC:3388] | 11863 | -0.563 | -0.3089 | Yes |
| 47 | LDLR | low density lipoprotein receptor [Source:HGNC Symbol;Acc:HGNC:6547] | 12009 | -0.595 | -0.2914 | Yes |
| 48 | MTOR | mechanistic target of rapamycin kinase [Source:HGNC Symbol;Acc:HGNC:3942] | 12047 | -0.605 | -0.2649 | Yes |
| 49 | LIF | LIF interleukin 6 family cytokine [Source:HGNC Symbol;Acc:HGNC:6596] | 12085 | -0.615 | -0.2379 | Yes |
| 50 | NTRK3 | neurotrophic receptor tyrosine kinase 3 [Source:HGNC Symbol;Acc:HGNC:8033] | 12157 | -0.632 | -0.2128 | Yes |
| 51 | DICER1 | "dicer 1, ribonuclease III [Source:HGNC Symbol;Acc:HGNC:17098]" | 12215 | -0.650 | -0.1857 | Yes |
| 52 | CLCF1 | cardiotrophin like cytokine factor 1 [Source:HGNC Symbol;Acc:HGNC:17412] | 12360 | -0.695 | -0.1632 | Yes |
| 53 | IL6 | interleukin 6 [Source:HGNC Symbol;Acc:HGNC:6018] | 12460 | -0.730 | -0.1355 | Yes |
| 54 | IL6ST | interleukin 6 cytokine family signal transducer [Source:HGNC Symbol;Acc:HGNC:6021] | 12505 | -0.749 | -0.1026 | Yes |
| 55 | NOTCH1 | notch receptor 1 [Source:HGNC Symbol;Acc:HGNC:7881] | 12555 | -0.776 | -0.0688 | Yes |
| 56 | HES1 | hes family bHLH transcription factor 1 [Source:HGNC Symbol;Acc:HGNC:5192] | 12676 | -0.849 | -0.0369 | Yes |
| 57 | CTNNB1 | catenin beta 1 [Source:HGNC Symbol;Acc:HGNC:2514] | 12866 | -1.119 | 0.0026 | Yes |
Table: GSEA details [plain text format]

  

Fig 2: GOBP\_REGULATION\_OF\_GLIAL\_CELL\_DIFFERENTIATION      
 Blue-Pink O' Gram in the Space of the Analyzed GeneSet

  

Fig 3: GOBP\_REGULATION\_OF\_GLIAL\_CELL\_DIFFERENTIATION: Random ES distribution      
 Gene set null distribution of ES for **GOBP\_REGULATION\_OF\_GLIAL\_CELL\_DIFFERENTIATION**
